# Rapidly enlarging dermatofibrosarcoma protuberans: a case report of atypical presentation with diagnostic complexities and reconstructive challenges

**DOI:** 10.1097/MS9.0000000000004682

**Published:** 2026-01-20

**Authors:** Syeda Rabiah Shahid, Maliha Khalid, Aminath Waafira

**Affiliations:** a Department of medicine, Shaikh Khalifa Bin Zayed Al-Nahyan Medical and Dental College, Karachi, Pakistan; b Department of medicine, Jinnah Sindh Medical University, Karachi, Pakistan; c School of Medicine, The Maldives National University, Malé, Maldives

**Keywords:** case report, dermatofibrosarcoma protuberans, flap reconstruction, rapid progression, wide local excision (WLE)

## Abstract

**Introduction and Importance:**

Dermatofibrosarcoma protuberans (DFSP) is a rare, low-grade dermal sarcoma known for local aggressiveness and high recurrence despite low metastatic potential. Early diagnosis and complete surgical excision with negative margins are critical for management. Reconstruction can be challenging, especially for large lesions. We report a rare case of rapidly enlarging DFSP over the lower chest–epigastric junction, complicated by diagnostic delays and complex reconstruction.

**Case Presentation:**

A 45-year-old man presented with a 4–5-month history of progressive midline chest swelling, low-grade fever, discomfort, and weight loss. Imaging revealed a vascular subcutaneous mass abutting the sternum. Fine needle aspiration cytology (FNAC) and biopsy remained inconclusive, delaying definitive diagnosis. Wide local excision with 3 cm margins created a 19 × 21 cm midline defect, requiring further extension based on frozen section. Reconstruction utilized a right paraumbilical perforator-based fasciocutaneous flap for primary closure. Final histopathology confirmed DFSP with clear margins and no high-grade transformation. Postoperative recovery was uneventful.

**Clinical Discussion:**

This case posed diagnostic and surgical challenges due to atypical presentation over the midline anterior abdominal wall, rapid enlargement of an otherwise indolent lesion, and inconclusive workup. Intraoperatively, the lesion’s vascularity and ill-defined margins prolonged surgery with significant blood loss. Multidisciplinary collaboration and intraoperative flexibility remained critical for achieving optimal oncologic and reconstructive outcomes.

**Conclusion:**

Early diagnosis, multidisciplinary planning, and tailored reconstructive strategies are vital in managing extensive, anatomically complex DFSP. This case highlights the importance of diagnosing rare presentations earlier with individualized, lesion-specific surgical approaches in achieving both functional and oncologic success.

## Introduction

Dermatofibrosarcoma protuberans (DFSP) is a rare, locally invasive, low-to-intermediate grade dermal and subcutaneous neoplasm, with an annual occurrence rate of 0.8–4.5 cases per million persons and accounts for approximately 1–6% of all soft tissue sarcomas^[1,2]^. Typically affecting young- to middle-aged adults with a slight male predominance, DFSP lesions most often involve the trunk, the proximal limbs and the head and neck region^[2,3]^. Its characteristic slow, infiltrative growth, and high rate of local recurrence confer an oxymoronic clinical profile- indolent yet aggressive. This necessitates treatment regimens to be centered around complete surgical excision with free margins^[4]^. Histologically, it is characterized by monomorphic spindle-shaped cells infiltrating subcutaneous fat with CD34 expression^[2,5,6]^. Although surgical excision remains the standard therapy, atypical presentations, diagnostic discrepancies due to inconclusive imaging, or treatment-seeking delays can complicate adequate management^[4,7]^. Reconstructive surgery after wide excision is essential, especially for large defects over functionally critical areas such as the anterior abdominal wall, and poses additional surgical challenges.

**HIGHLIGHTS**

- *Rapid growth and diagnostic delay*: An unusually fast-enlarging DFSP in the lower chest–epigastric region led to prolonged diagnostic work-up (over 2 months), with nondiagnostic FNAC and equivocal immunohistochemical findings delaying definitive treatment.
- *Intraoperative complexity*: Ill-defined tumor margins, significant vascularity, and positive frozen sections at deep margins necessitated prolonged surgery, intraoperative re-excision, and meticulous hemostasis before reconstruction.
- *Tailored reconstructive strategy*: A large 19 × 21 cm defect was successfully closed using a paraumbilical perforator-based fasciocutaneous flap, with staged tissue mobilization on the ischemic side to ensure both oncologic clearance and optimal functional and aesthetic outcomes.

Here, we report a case of rapid enlargement of an otherwise indolent DFSP tumor, presenting at an unusual location – over the midline lower chest–epigastric junction – with significant vascularity and localized protrusion as well as invasion, contributing to diagnostic delays and reconstructive complexities. This atypical behavior underscores the importance of early recognition and lesion-specific surgical planning, offering additional insights into the limited literature on unusually rapid progression of DFSP and its management. Multidisciplinary coordination is essential for both oncologic clearance and functional reconstruction, particularly when anatomically sensitive areas are involved.

This case report follows the principles of the SCARE guidelines, attached as Supplemental Digital Content 1, available at: http://links.lww.com/MS9/B79, to ensure comprehensive data reporting for proper, precise, and patient-centered management of unusual cases with aggressive behaviors^[8]^.

## Case presentation

A case of a 45-year-old gentleman, with no known comorbidities, a 10-pack-year smoking history, and a 15-year prior history of successfully treated pulmonary tuberculosis with no current intake of any medications, presented with a progressively enlarging swelling, involving the central lower chest and upper epigastric regions of the anterior abdominal wall.

**Figure 1.**
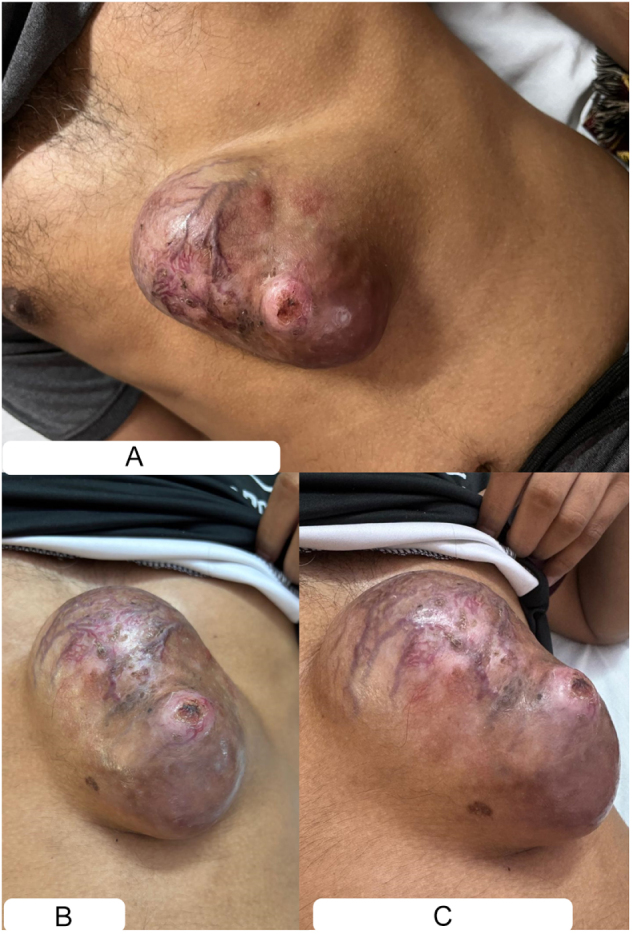
Preoperative clinical images of the lower chest–epigastric mass: (A and B) superior view showing midline pear-shaped swelling, demonstrating overlying shiny violaceous skin; and (C) lateral view showing close-up of punctum and prominent vascularity.

Approximately 7 months prior, the patient noticed a small nodule in the lower midline chest, which expanded significantly over the past 4–5 months into a sizable mass (Fig. 1). He reported mild pain and discomfort, relieved in a supine position, undocumented nocturnal low-grade fever, and weight loss – evidenced by loosened clothes.

## Examination findings

On examination, an 11 × 10 cm pear-shaped midline swelling was observed at the junction of the anterior lower chest and upper epigastric region (Fig. 1). The lesion was firm, immobile, noncompressible, and non-fluctuant, with a visible punctum and a shiny, slightly violaceous overlying skin with prominent intrinsic vascularity. On palpation, mild tenderness and warmth were observed, but signs of overt local infection, satellite lesions, or regional lymphadenopathy were absent. The patient appeared clinically and vitally stable. Based on these findings, multiple differential diagnoses were considered including, epidermoid cyst (central punctum, superficial location), abscess (tenderness, low-grade fever), desmoid tumor (firm, abdominal wall mass), hemangioma/vascular malformation (prominent vascularity, violaceous skin), spindle cell neoplasms (rapid growth, firm consistency), lipoma (subcutaneous mass, atypical rapid growth), unusual site of tuberculous lymphoma (history of TB, systemic symptoms), and DFSP (firm, violaceous lesion). Thereby, further investigational evaluation was warranted.

## Investigations

**Figure 2.**
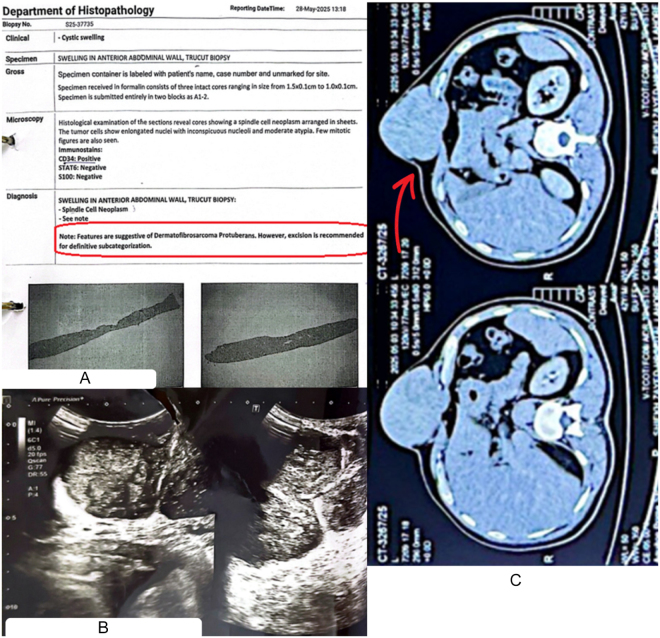
Investigations: (A) Tru-cut biopsy showing spindle cell neoplasm with moderate atypia, immunohistochemistry positive for CD34 and negative for STAT6 and S100, consistent with DFSP; (B) ultrasonography abdomen, demonstrating a heterogeneous, multiloculated subcutaneous mass with marked vascularity; and (C) CT contrast chest/abdomen (coronal view), showing a well-marginated, solid subcutaneous mass (11.6 cm) with intense enhancement and focal necrosis, abutting the sternum but not invading deeper structures.

Ultrasonography of the mass revealed a heterogeneous, multiloculated, subcutaneous mass with marked vascularity, measuring up to 10 × 5.9 cm, as shown in Figure 2. Aggressive vascularity was further evidenced by color Doppler imaging. Subsequent contrast-enhanced CT thorax and MRI confirmed a well-marginated, solid mass in the subcutaneous midline lower chest, measuring up to 11.6 cm, with intense enhancement and focal areas of necrosis, thereby ruling out multiple differentials including an epidermoid cysts, lipomas, and infectious abscesses (Fig. 2). The lesion was limited to the subcutaneous tissue, abutting but not invading the underlying sternum, with no deeper intra-abdominal extensions or metastasis.

An initial Ultrasonography (USG)-guided fine needle aspiration cytology (FNAC) was nondiagnostic, showing only blood and degraded cells. Tru-cut biopsy revealed a spindle cell neoplasm with moderate atypia, arranged in sheets with elongated nuclei and inconspicuous nucleoli (Fig. 2). Immunohistochemistry was only slightly positive for CD34, while STAT6 and S100 were negative, ruling out most of the remaining differentials, including vascular tumors and other soft tissue sarcomas. However, suspicion remained between a definitive diagnosis of a dermoid cyst, spindle cell tumors, and DFSP, even after 2 long months of extensive diagnostic testing, owing to inconclusive biopsy results (Table 1).

**Table 1:** Timeline of events

Event	Date/duration	Details
Initial nodule appearance	~7 months before presentation	Small midline chest nodule noted
Rapid enlargement	4–5 months before presentation	Pain, skin changes, systemic symptoms
Hospital presentation	17 April 2025	OPD visit; 11 × 7 cm lesion on examination
Ultrasonography cystic mass	21 April 2025	10 × 5.9 cm cystic lesion; thick intrinsic vascularity
Ultrasound abdomen and color Doppler	24 April 2025	6.5 × 3.8 × 3.4 cm mass; Aggressive flow on color Doppler
CT chest, abdomen, and pelvis with IV contrast	7 May 2025	Well-defined margins; intrinsic vascularity and deep invasion involving musculature
USG-guided FNAC	14 May 2025	Blood and degenerate cells only; excision recommended
Hospital admission	22 May 2025	Cystic swelling on anterior abdominal wall
Tru-cut biopsy	24 May 2025	Spindle cell neoplasm; STAT6 and S100 −ive CD34 +ive
Contrast MRI chest and abdomen	30 May 2025	Large lesion abutting sternum; no metastasis
Wide local excision	4 June 2025	Mass excised, creating a 19 × 21 cm defect; flap reconstruction
Secondary closure	11 June 2025	Local tissue rearrangement for flap compromise
Hospital discharge	19 June 2025	No signs of infection, discomfort or hemorrhagic discharge; advised for follow-up visits and regular dressing
Follow-up	1 week, 2 weeks, and 6 months	Stable wound healing, no recurrence, satisfactory outcome

CT, computed tomography; FNAC, fine needle aspiration cytology; MRI, magnetic resonance imaging.

## Treatment and outcome

**Figure 3.**
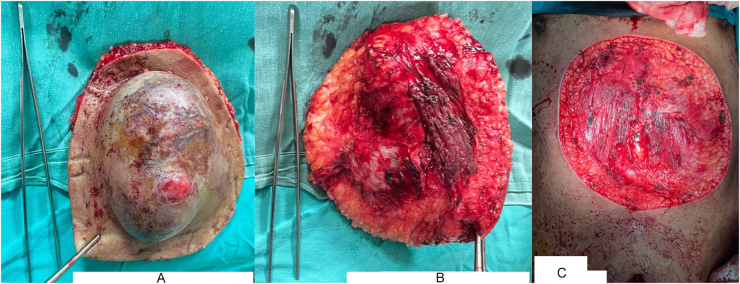
Gross specimen and resultant defect: (A) anterior view of excised mass (11 × 10 × 7.9 cm); (B) posterior view; and (C) midline defect post-excision measuring 19 × 21 cm.

A wide and deep local excision under general anesthesia was performed, and a mass measuring 11.0 × 10.0 × 7.9 cm was excised, producing a 19 × 21 cm defect extending from the xiphisternum to 10 cm above the umbilicus, reaching the rectus muscle at the basal margin (Fig. 3).

**Figure 4.**
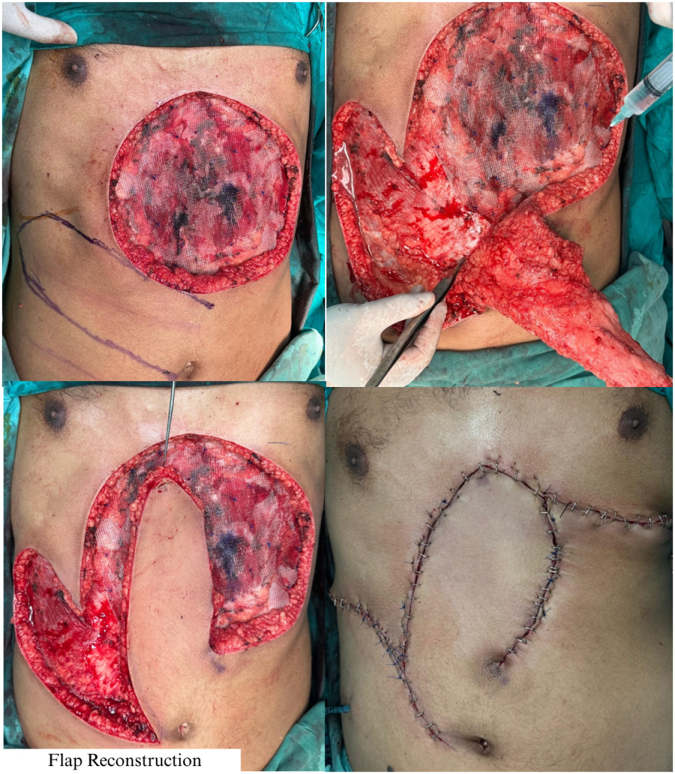
Reconstructive procedure. Right paraumbilical perforator-based fasciocutaneous flap partially closing the defect, with left-sided tissue transposition for wound approximation.

Despite a 3 cm safety margin, the intraoperative frozen section analysis of the excised specimen showed the presence of tumor at the base, consisting of spindle cells infiltrating fat with 5 mitoses per 10 high-power fields without high-grade transformation. Thus, an additional extension of the deep margins was obtained and confirmed free of tumor before reconstruction was performed. Rapid progression, considerably large size, reconstructive requirements, deep local extension into the fascia and marked vascularity of the lesion necessitated an immediate wide local excision (WLE) to achieve clear margins, making Mohs micrographic surgery unfeasible. Due to the extensive tissue and blood loss resulting in a significantly deep defect in a vulnerable area, reconstruction was performed using a right paraumbilical perforator-based fasciocutaneous flap, as shown in Figure 4. While primary closure was possible on the right side, on the left side, tissue transposition and mobilization were deemed necessary for wound approximation.

## Post-treatment outcomes and challenges

In the immediate postoperative period, antibiotic care was initiated with cefoperazone–sulbactam, gentamicin, and linezolid injections. Vacuum-assisted closure (VAC) therapy was maintained on a continuous setting; however, the foam dressing remained persistently soaked with hemorrhagic discharge.

**Figure 5.**
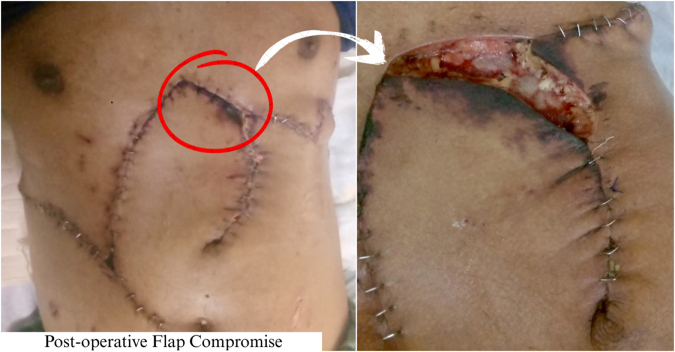
Postoperative flap compromise. Mild ischemic changes at the upper flap margin on postoperative day 4, showing purplish-black discoloration and slight delay in capillary refill with no signs of active infection, were managed with secondary tissue rearrangement.

Although flap viability was initially preserved, by postoperative day 4, a purplish-black discoloration developed at the upper flap margin with slight delays in capillary refill, suggestive of mild ischemia (Fig. 5). A secondary surgical procedure, employing local tissue rearrangement, was performed to achieve closure of the left wound edge.

Final histopathology confirmed DFSP, with low mitotic index, clear margins on extended resection and absence of necrosis or transformation. The patient was vitally stable and thoroughly counselled. Following the second procedure, the VAC therapy was discontinued; however, the postoperative antibiotic regimen was extended until the date of discharge, 1 week later. Follow-up at weeks 1 and 2 demonstrated a clean, healing wound without signs of infection. The patient was advised to perform twice-daily dressing changes with saline irrigation. At the 6-month mark, the patient was pleased with the outcome, had recovered to normal functioning, and showed no evidence of recurrence, with the surgical scars properly healed.

## Discussion

DFSP is classified as a rare, locally aggressive yet indolent cutaneous sarcoma with high recurrence potential^[9,10]^. The surgical and reconstructive management must be tailored depending upon tumor size and depth, anatomical location, proximity to vital structures, and the need for functional and aesthetic preservation^[11–15]^. Although these tumors predominantly affect the head and neck regions, the posterior trunk, the shoulders, and the limbs, recent cases have reported anterior abdominal wall presentations. However, these cases presented with painless and smaller lesions, with minimal skin changes, no tortuous vascularity, and a prolonged period of enlargement spanning over several months to years^[14–17]^. In contrast, our patient presented with a rapidly enlarging, violaceous lesion with a central punctum and tortuous vascularity, over the anterior midline, at the lower chest and upper epigastrium junction, posing significant diagnostic ambiguity between DFSP^[15]^, desmoid tumors^[18]^, epidermoid cysts^[19]^, hemangiomas, and other spindlecell neoplasms. Moreover, due to the patients’ prior history of tuberculosis, additional diagnostic confusion was faced with similar cutaneous manifestations of extranodal lymphomas^[20,21]^. Additionally, the initial FNAC results were nondiagnostic and radiologic features remained indeterminate, reinforcing challenges described in prior literature^[22,23]^. Two months of thorough imaging and extensive histopathological evaluation, including immunohistochemistry, showing slight CD34 positivity with negative STAT6 and S100, helped exclude other soft tissue sarcomas; however, the definitive diagnosis of DFSP remained elusive until eventually established. These diagnostic delays are not uncommon, thereby underscoring the need for histopathological and immunohistochemical evaluation for unusual soft tissue masses for accurate diagnosis and appropriate surgical planning^[24,25]^.

The mainstay treatment for DFSP remains complete surgical excision with histologically negative margins. While multiple surgical techniques have been employed, WLE with 2–3 cm margins or Mohs micrographic surgery with the added benefit of lower recurrence rates remains the standard approach^[4,12,26]^. In our case, Mohs Micrographic Surgery (MMS) was deemed unfeasible owing to the tumor’s rapid progression, large size, high intrinsic vascularity, and extensive tissue invasion^[27,28]^. Thus, a WLE with 3 cm safety margins was performed, which posed significant surgical and reconstructive challenges, particularly owing to the close proximity of the lesion to structurally vulnerable areas and relatively inelastic overlying skin. Intraoperative frozen section analysis identified residual tumor at the deep margin, necessitating prompt re-excision to ensure tumor-free margins and optimize oncologic outcomes, given DFSP’s notorious proclivity for local recurrence^[9,16,26]^. This, however, resulted in a substantial 19 × 21 cm midline defect in the anterior abdominal wall extending from the xiphisternum to approximately 10 cm above the umbilicus and down to the rectus sheath and muscles.

A functionally and aesthetically preserving reconstruction was warranted to ensure both structural durability and flexibility to accommodate respiratory motion, preserve abdominal wall integrity, and prevent ischemic necrosis. A paraumbilical perforator-based fasciocutaneous flap was used due to its robust vascular supply, local availability, and adaptability for large anterior wall defects, minimizing donor-site morbidity^[29–31]^. The right side allowed primary closure, while the left required tissue transposition due to limited local laxity.

Early postoperative ischemic changes in the superior flap margin were promptly recognized and corrected with local tissue rearrangement, demonstrating the importance of meticulous postoperative monitoring of temperature, discoloration, and capillary refill time, especially within the critical first 72 h^[32–34]^.

VAC dressing system supported wound healing, minimized dead space, and reduced risk of infections and seromas, although careful observation was mandated to manage hemorrhagic discharge^[35,36]^.

Appropriate conservative measures, including extensive prophylactic antibiotic regimens, close monitoring, and timely scheduled subsequent procedures, were able to hemodynamically stabilize the wound, eventually leading to proper healing with no observed signs of infection at follow-up visits.

The reconstruction plan was designed to accommodate potential future interventions such as radiotherapy, upon the detection of recurrence or microscopic residual disease. Flap-based reconstructions, in contrast to skin grafts, offer superior healing in terms of adjuvant radiotherapy with minimal risk of radiation-induced necrosis as well as easy access to the tumor site if recurrence is to be observed^[37,38]^. Although this case provides valuable insights for managing challenging cases of rapidly enlarging DFSP in anatomically complex sites and the need for a multidisciplinary approach, several limitations must be acknowledged. As a case report of a single patient, generalizability may be reduced. The lack of long-term follow-up, beyond the 6-month mark of postoperative period, further deteriorates the robustness of oncologic success.

## Conclusion

DFSP management requires early recognition, tailored surgical excision with clear margins, and individualized reconstruction employing a multidisciplinary approach, involving surgical oncology, histopathology, and plastic surgery experts. Regional perforator-based flaps offer a reliable solution for anterior trunk defects while maintaining oncologic safety, functional integrity, and aesthetic outcomes. This case reinforces the importance of precision, adaptability, and patient-centered care. While neoadjuvant measures and reconstructive therapies continue to emerge and improve outcomes in complex DFSP presentations, the experience from this case further supports the evolution of early planning for optimal patient recovery, aesthetic preservation, and long-term oncologic control.

## Data Availability

No data were generated for this manuscript.

## Research registration unique identifying number (UIN)

(1) Name of the registry: N/A. (2) Unique Identifying number or registration ID: N/A. (3) Hyperlink to your specific registration (must be publicly accessible and will be checked): N/A.
